# Acetozone in Typhoid Fever

**Published:** 1902-08

**Authors:** G. H. Westinghouse

**Affiliations:** Buffalo, N. Y.; Visiting Physician at the Buffalo Hospital Sisters of Charity


### CLINICAL REPORTS.

### Acetozone in Typhoid Fever.

By G. H. WESTINGHOUSE, M. D., Buffalo, N. Y.
Visiting Physician at the Buffalo Hospital Sisters of Charity.

AS THE writer has had a somewhat extensive experience in the use of acetozone (benzoil-acetyl-peroxide) in the treatment of typhoid fever, he thinks it may be of interest to the profession at large to briefly review a few of the more severe or complicated cases, and to add a few comments bearing upon the action of this new therapeutic agent. Unfortunately, many of the bed-side records of the patients have been lost, rendering impossible a complete report of many of the cases thus treated.

Altogether forty cases of typhoid were under my care at the hospital during the summer of 1901. Complications were many and varied, and the patients were admitted during all stages of the disease. There were, however, no hemorrhages, and not a single fatality. The following statements are presented of the results obtained during the treatment:

Case I.—F.R., aged 14 years; admitted to the hospital July 27, 1901, this being the fifth day of the disease; temperature when admitted, 101°. Acetozone treatment was begun immediately. The temperature began to subside at once, becoming normal on the fourteenth day. On the nineteenth day endocarditis developed, with a rise in temperature to 103 1-5°, this continuing until the twenty-second day, when the temperature again subsided. On the twenty-fourth day phlebitis of the saphenous vein developed with a rise in temperature 101 2-5°, which gradually subsided. No hemorrhages occurred and recovery was complete, the patient being discharged on the forty-second day.

Case II.—P.S., aged 37 years, with a temperature of 102 1-5°, was admitted to the hospital August 28, 1901, this being the seventh day of the disease. Acetozone treatment was begun immediately, with a gradual subsidence of temperature. Soon after this, pneumonia with kidney complications developed, with a rise in temperature which gradually declined and was followed by complete recovery. The patient was discharged on the fifty-eighth day of the disease.

Case III.—J. M., aged 12 years. Entered hospital on the eighth day of the disease with a temperature of 103 1-5°. Acetozone treatment was begun immediately; a gradual reduction in temperature followed, becoming normal on the fourteenth day and remaining practically normal until his discharge. which occurred on the fortieth day of the disease. Convalescence was prolonged on account of complications which followed typhoid symptoms.

Case IV.—W. R., aged 9 years. Admitted to the hospital on the fourth day of the disease, with a temperature of 103 4-5°. Acetozone treatment was begun immediately, and temperature became normal on the fourteenth day. Owing to complications which developed following the reduction of temperature to normal on the fourteenth day, the convalescence of this patient was somewhat prolonged; but he was finally discharged with complete recovery about the thirty-fifth day.

Case V.—Mrs. C., aged 30. Admitted to hospital on tenth day of disease, with temperature of 103°. Acetozone treatment began immediately, with reduction of temperature to normal on the fourteenth day. This case is remarkable from the fact that at the climax of the disease, collapse developed. Subcutaneous injections of acetozone solution raised to the normal alkalinity were injected under the breasts on two occasions; twenty-four hours intervened between the injections; another injection of 500 c.c. acetozone solution with 500 c.c. normal salt solution, after another interval of twenty-four hours. A noticeable improvement followed immediately after the injection of the acetozone solution. This patient went on to perfect recovery, and was discharged about the thirty-eighth day of the disease.

Case VI.—A. W., aged 16 years, was admitted to the hospital August 12, 1901, on the tenth day of the disease; temperature, 104 1-2°; patient muttering in delirium; abdomen enormously distended; condition decidedly toxic. The use of acetozone solution was begun immediately; the temperature declined to the normal on the fourteenth day of the disease and continued normal until the patient's discharge, on the twenty-eighth day. Recovery was uncomplicated and uneventful.

Case VII.—C. K., aged 24 years, was admitted on the ninth day of the disease; temperature, 102 1-5°. Treatment by acetozone solution was begun immediately. The temperature reached the normal on the fourteenth day of the disease; convalescence was rapid and the patient was discharged from the hospital on the twenty-fifth day after admission. No complications occurred and recovery was complete.

In order not to prolong this article, the writer will conclude by saying that the convalescence of the patients was rapid, but they were retained in the hospital a considerable length of time to insure against a relapse. Under the use of acetozone the characteristic fetor of typhoid stools completely disappeared in two or three days; the usual relaxed condition of the bowel had wholly subsided by this time, rendering the use of either enema or laxative unnecessary.

Not the least useful effect of the acetozone was the diuresis developed when the aqueous solution of the antiseptic was taken in quantities sufficient to render the alimentary canal aseptic. This diuretic effect was marked, one patient having voided no less than 112 ounces of urine in twenty-four hours. A corresponding reduction of typhoid symptoms followed; tympanitis and delirium disappeared. The diagnosis of typhoid fever was confirmed, in all cases, in the laboratory by Widal's reaction, Ehrlich's test, and by blood count.

It is unnecessary to say anything further than that acezotone, as an intestinal antiseptic, is unequaled by anything I have ever employed. A complete subsidence of all the bowel symptoms followed in every case of typhoid within a few days after beginning its use. The application of the antiseptic consisted, in most cases, in simply allowing the patient to drink the saturated solution, *ad libitum*; or, in other words, substituting this solution for all other liquids, and urging the patient to partake of it freely where the natural craving was not sufficient to insure the consumption of considerable quantities.

NOTE.—The antiseptic above referred to, benzoil-acetyl-peroxide, was originally supplied by Messrs. Parke, Davis & Co., of Detroit, Mich., under the name of benzozone. The manufacturers have recently announced that in order to avoid confusion through a resemblance of this term to the name of another antiseptic placed upon the market some years ago, it has been decided to employ the name acetozone, instead of benzozone.

